# Outcome and Safety of Anterograde and Retrograde Single-Balloon Enteroscopy: Clinical Experience at a Tertiary Medical Center in Taiwan

**DOI:** 10.1371/journal.pone.0161188

**Published:** 2016-08-22

**Authors:** Meng-Chiung Lin, Peng-Jen Chen, Yu-Lueng Shih, Hsin-Hung Huang, Wei-Kuo Chang, Tsai-Yuan Hsieh, Tien-Yu Huang

**Affiliations:** 1 Division of Gastroenterology, Department of Internal Medicine, Tri-Service General Hospital, National Defense Medical Center, Taipei, Taiwan; 2 Department of Internal Medicine, Taichiung Armed Forces General Hospital, Taichiung, Taiwan; 3 Taiwan Association for the Study of Small Intestinal Diseases, Taoyuan, Taiwan; Medizinische Fakultat der RWTH Aachen, GERMANY

## Abstract

Single-balloon enteroscopy (SBE) is designed for identifying possible small bowel lesions with balloon-assisted enteroscopy that allows deep intubation of the intestine. However, data regarding the outcome and safety of SBE remain limited. We conducted this study to evaluate the outcome and safety of anterograde and retrograde SBE approaches. This retrospective review from a tertiary medical center in Taiwan included endoscopic reports and chart data from 128 patients with 200 anterograde and retrograde procedures from September 2009 to November 2014. In this study, the most common indication for both anterograde and retrograde SBE was obscure gastrointestinal bleeding (64.4% vs. 60.6%). There were no significant differences between anterograde and retrograde approaches in terms of the diagnostic yield (69.3% vs. 52.5%) and intervention rate (23.8% vs. 17.2%). The procedure time was shorter for anterograde SBE than for retrograde SBE (68.1 ± 23.9 vs. 76.8 ± 27.7 min, *P* = 0.018). In addition, among the subgroup of patients with obscure gastrointestinal bleeding, the most common etiologies for those in different age-groups were angiodysplasia (≥ 65 years), non-specific ulcers (30–64 years), and Meckel’s diverticulum (< 30 years). The major complication rate during the study was 1.5%; the rate of asymptomatic hyperamylasemia was higher for patients who underwent anterograde SBE than for those who underwent retrograde SBE (13.9% vs. 2%, *P* = 0.005). The outcome and safety of anterograde and retrograde SBE are similar. However, anterograde SBE has a shorter procedural time and a higher rate of asymptomatic hyperamylasemia.

## Introduction

Before the year 2000, small bowel disease was considered rare, which was a consequence of the lack of adequate small-bowel diagnostic procedures. In 2001, the introduction of capsule endoscopy provided direct visualization of the whole small intestinal mucosa [1]. However, therapeutic procedures and tissue sampling are not possible with capsule endoscopy. Later, the development of double-balloon enteroscopy (DBE) enabled diagnostic and therapeutic procedures in the small intestine [2]. In 2007, the invention of single-balloon enteroscopy (SBE) simplified the DBE procedure by allowing manipulation with a single balloon. Balloon-assisted enteroscopy is a useful tool for the diagnosis and treatment of small bowel lesions [3]. The majority of data concerning the outcomes and safety of balloon-assisted enteroscopy have been obtained from studies using DBE, and data obtained from studies using SBE are limited [4–6]. In addition, comparisons of anterograde and retrograde SBE for outcome and safety are lacking [7]. Therefore, more studies are required to verify the reliability of SBE with respect to outcome and safety. The aims of this study were to emphasize the indication, diagnostic, and intervention yields, complications, and outcomes of both anterograde and retrograde SBE in a tertiary medical center in Taiwan.

## Methods and Materials

A total of 200 consecutive anterograde and retrograde procedures were performed in 128 patients, who underwent SBE for suspected small bowel lesions, at the Taiwanese Tri-Service General Hospital between September 2009 and December 2014. Within this cohort, 80 patients received a uni-directional SBE, 33 received bi-directional SBE, and 14 received more than 2 SBE procedures. After informed of the benefits and potential risks of SBE, all the patients signed an informed consent before SBE. The need for patient consent in this study was waived because patient information was anonymized and deidentified prior to analysis. This retrospective study protocol was conducted according to the principles of the Declaration of Helsinki and was approved by the institutional review board at the Tri-Service General Hospital (TSGHIRB: 1-104-05-035) in Taiwan.

The characteristics of patients who underwent SBE, including age, sex, and body mass index, were recorded according to patient medical charts. The indications for SBE included obscure gastrointestinal bleeding (OGIB), unexplained abdominal pain, chronic diarrhea, small bowel tumor, suspected inflammatory bowel disease, and removal of a small intestinal foreign body. For anterograde SBE, fasting ≥ 8 hours was required prior to the procedure. For retrograde SBE, bowel preparation was performed with sodium phosphate or polyethylene glycol solution. The initial insertion route for SBE (oral or anal) was dependent on the judgment of clinical presentation (melena vs. hematochezia) and images (abdominal tomography, small intestinal series, red blood cell [RBC] scan, and Meckel’s scan). All SBE procedures were performed by an experienced endoscopist using an Olympus single-balloon enteroscope (SIFQ260; Olympus Optical, Tokyo, Japan). Carbon dioxide was used for insufflation during the procedure to decrease post-procedure abdominal fullness. Before SBE, hyoscine-N-butylbromide, meperidine, and midazolam were prescribed on demand. Provided that no contraindications existed, hyoscine-N-butylbromide (10 mg), meperidine (25 mg), and midazolam (2.5 mg) were administered intravenously prior to SBE; repeat administration using the same doses was performed during SBE. A cap was attached at the tip of the enteroscope to improve the visual field and mucosal hooking during the procedure. Intervention procedures were defined as those including endoscopic therapy. Tattoo marking with 0.5 mL Spot solution (GI Supply, Camp Hill, PA, USA) was performed at the deepest area of advancement. In cases where SBE did not detect the symptom-related lesion and the patient agreed to further intervention, the opposite approach was performed. If the tattoo marking area, made during the initial approach, was observed via the opposite approach, the whole small intestinal examination was considered complete. Procedure failure was defined as the inability to progress the scope tip beyond the ligament of Treitz in the anterograde approach, or an inability to reach 30 cm beyond the ileocecal valve in the retrograde approach (except in cases with disease-related stricture or stenosis). SBE procedures were performed according to the SBE system instructions provided by Olympus. One day after the SBE procedure, the serum amylase level was checked.

The general characteristics of patients were analyzed. All data were presented as the mean ± standard deviation (SD) for continuous variables, or the number (percentage) for categorical variables. Statistical analysis was performed using PASW statistics software, version 18 (IBM Co., Somers, NY, USA). Continuous variables were compared using Student’s *t*-test, and categorical variables were compared using chi-square and Fisher’s exact tests. All reported *P* values are 2-tailed, and *P* < 0.05 was considered statistically significant.

## Results

A total of 200 consecutive SBE procedures were performed in the 128 patients who were enrolled in this study (Table 1). Patients were scheduled for diagnostic and/or therapeutic SBE. Indications for SBE were as follows: OGIB in 125 (62.5%) procedures, unexplained abdominal pain in 25 (12.5%), small intestinal tumor in 25 (12.5%), Crohn’s disease activity assessment in 9 (4.5%), intestinal obstruction in 7 (3.5%), chronic diarrhea in 6 (3.0%), and image abnormality in 6 (3%). Other indications included foreign body extraction in 1 patient and nasojejunal tube placement in another case. In patients with chronic diarrhea, the retrograde approach was more common than the anterograde approach (6 vs. 0 patients, *P* = 0.012). The diagnostic rate of SBE was 69.3% for the anterograde approach and 52.5% for the retrograde approach, with an overall diagnostic rate of 61%. In total, 52.5% of procedures (103) were performed in cases with newly diagnosed disease. The median age of the 128 patients (66 men and 62 women) who underwent SBE procedures was 65 years (range, 14–94 years). One-hundred-and-one procedures (50.5%) were performed via the anterograde approach, and 99 procedures (49.5%) were performed via the retrograde approach. The mean approach time of the procedures was 72.4 minutes (72.4 ± 26.2 min). The mean approach time was 68.1 minutes (68.1 ± 23.9 min) for anterograde SBE, and 76.8 minutes (76.8 ± 27.7 min) for retrograde SBE. The duration of the approach with retrograde SBE was longer than that with anterograde SBE (76.8 min vs. 68.1 min, *P* = 0.018). However, there were no other significant differences among the basic characteristics of patients, and the diagnostic and intervention rates, between the anterograde and retrograde SBE approaches. The most common endoscopic finding was angiodysplasia (15.2%, Table 2). Although the detection rate of the anterograde approach was higher than that of the retrograde approach (18.9% vs. 11.6%), there were no significant differences between these approaches in the detection rate for angiodysplasia (*P* = 0.158).

**Table 1:** Clinical characteristics of patients receiving single balloon enteroscopy.

Characteristics	Total SBE (n = 200)	Anterograde SBE (n = 101)	Retrograde SBE (n = 99)	P value
	N (%)	N (%)	N (%)	
Age ± SD, years	58.22 ± 20.69	60.42 ± 20.27	55.98 ± 19.95	0.130
Sex (M, %)	101 (50.5)	54 (53.5)	47 (47.5)	0.397
BMI	22.94 ± 3.29	22.81 ± 3.29	23.08 ± 3.31	0.607
Indications				
OGIB	125 (62.5)	65 (64.4)	60 (60.6)	0.584
Unexplained abdominal pain	25 (12.5)	13 (12.9)	12 (12.1)	0.873
IBD	9 (4.5)	3 (3.0)	6 (6.1)	0.292
Chronic diarrhea	6 (3.0)	0 (0.0)	6 (6.1)	0.012
Intestinal obstruction	7 (3.5)	5 (5.0)	2 (2.0)	0.260
Small intestinal tumor	17 (8.5)	7 (6.9)	10 (10.1)	0.422
Image abnormality	6 (3.0)	4 (4.0)	2 (2.0)	0.421
Others	5 (2.5)	4 (4.0)	1 (1.0)	0.181
Diagnostic rate	122 (61.0)	70 (69.3)	52 (52.5)	0.715
Intervention rate	41 (20.5)	24 (23.8)	17 (17.2)	0.482
Total approach time (mean ± SD, min)	72.4 ± 26.2	68.1 ± 23.9	76.8 ± 27.7	0.018

SBD: single balloon enteroscopy; SD: standard deviation; BMI: body mass index; OGIB: obscure gastrointestinal bleeding; and IBD: inflammatory bowel disease

**Table 2:** Total endoscopic findings of single balloon enteroscopy procedures.

Characteristics	Total SBE (n = 190*) N (%)	Anterograde SBE (n = 95) N (%)	Retrograde SBE (n = 95) N (%)	P value
Enteroscopy findings				0.148
Angiodysplasia	29 (15.3)	18 (18.9)	11 (11.6)	0.158
Diverticulum	8 (4.2)	3 (3.2)	5 (5.3)	0.470
Benign tumors	10 (5.3)	1 (1.1)	9 (9.5)	0.009
Malignant tumors	17 (8.9)	9 (9.5)	8 (8.4)	0.799
Meckel's diverticulum	3 (1.6)	0 (0.0)	3 (3.2)	0.081
IBD	10 (5.3)	4 (4.2)	6 (6.3)	0.516
Ulcer bleeding	17 (8.9)	9 (9.5)	8 (8.4)	0.799
Non-specific lesions	13 (6.8)	6 (6.3)	7 (7.4)	0.774
Others	23 (12.1)	14 (14.7)	9 (9.5)	0.266
Negative	60 (30.1)	31 (32.6)	29 (30.5)	0.875

* Total SBE procedures excluded 10 procedures performed during follow-up.

SBE: single-balloon enteroscopy; and IBD: inflammatory bowel disease

The total intervention rate of SBE was 20.5% (anterograde 23.3%, retrograde 17.2%; Table 1). The most common interventional procedure was hemostasis. The hemostasis rate was higher in anterograde SBE than that in retrograde SBE (22.2% vs. 11.9%, *P* = 0.069, Table 3). Therapeutic interventions were performed according to the different clinical conditions, which included argon-plasma coagulation (APC), polypectomy, hot biopsy, diluted epinephrine injection therapy, and foreign body extraction. The most common methods for hemostasis were APC and endoscopic hemoclip placement (Table 3). In 1 patient with recurrent small intestinal lipomatosis, a total of 4 polypectomies with unroofing were performed. Procedure failure occurred for 1 patient who underwent the anterograde approach and 3 patients who underwent the retrograde approach.

**Table 3:** Endoscopic intervention of patients receiving single balloon enteroscopy

Characteristics	Total SBE (n = 200) N (%)	Anterograde SBE (n = 101) N (%)	Retrograde SBE (n = 99) N (%)	P value
Hemostasis	34 (17)	22 (22.2)	12 (11.9)	0.069
APC	21 (10.5)	12 (11.9)	9 (9.1)	0.520
Hemoclip	11 (5.5)	8 (7.9)	3 (3.0)	0.248
Diluted epinephrine	9 (4.5)	9 (8.9)	0 (0.0)	0.002
Hot biopsy	5 (2.5)	3 (3.0)	2 (2.0)	0.667
Removal of foreign body	1 (0.5)	1 (1.0)	0 (0.0)	0.321
Polypectomy	6 (3.0)	1 (1.0)	5 (5.1)	0.092

SBE: single balloon enteroscopy; APC: argon plasma coagulation

The leading indication for SBE was OGIB (62.5% of all SBE procedures). OGIB patients were stratified into 3 age groups (< 30 years, 30–65 years, > 65 years) in order to investigate the common etiologies of OGIB in different age groups. As shown in Table 4, the most common etiology of patients with OGIB who were < 30 years, 30–65 years, and > 65 years were Meckel’s diverticulum, non-specific ulcer, and angiodysplasia, respectively.

**Table 4:** Common etiologies of patients with obscure gastrointestinal bleeding (N = 85)

	< 30 age (N = 11)	30–65 age (N = 26)	> 65 age (N = 48)
1^st^	Meckel's diverticulum (3, 17.7%)	Non-specific ulcer (7, 26.9%)	Angiodysplasia (20, 27.0%)
2^nd^	Non-specific ulcer (2,11.8%)	Tumor (6, 23.1%)	Non-specific ulcer (5, 13.5%)
3^rd^	Angiodysplasia (1, 5.9%), Tumor (1, 5.9%), Diverticulum (1, 5.9%)	Angiodysplasia (5, 19.2%)	Tumor (4, 5.5%), Diverticulum (4, 5.4%)

SBE: single balloon enteroscopy; OGIB: obscure gastrointestinal bleeding; IBD: inflammatory bowel disease

For the evaluation of SBE safety, major and minor complications were assessed in detail (Table 5). In the therapeutic approach with SBE, the only major complication associated with the anterograde SBE approach was acute pancreatitis following biopsy and hemostasis for 1 patient with a duodenal tumor. The other 2 major complications occurred during SBE via the retrograde approach. No aspiration pneumonia was detected. One patient with inverted Meckel’s diverticulum mimicking a polyp developed perforation after polypectomy, and another case with lipomatosis at the distal ileum had delayed bleeding after unroofing resection. The overall major complication rate was 1.5%; there were no significant differences in major complications between the anterograde and retrograde approaches. Mild abdominal fullness and discomfort were the most common minor complications of SBE, which resolved spontaneously without medical intervention. Sixteen patients (8.0%) developed asymptomatic hyperamylasemia after receiving SBE (anterograde vs. retrograde SBE: 13.9% vs. 2.0%, *P* = 0.005).

**Table 5:** Overall complications associated with single-balloon enteroscopy procedures.

Complications		Total SBE (N = 200) N (%)	Anterograde SBE (N = 101) N (%)	Retrograde SBE (N = 99) N (%)	*P value
Category	Complication				
Major					
	Pancreatitis	1 (0.5)	1 (1.0)	0 (0.0)	0.321
	Bleeding	1 (0.5)	0 (0.0)	1 (1.0)	0.311
	Perforation	1 (0.5)	0 (0.0)	1 (1.0)	0.311
Minor					
	Hyperamylasemia	16 (8.0)	14 (13.9)	2 (2.0)	0.005

*Pearson chi-square test. SBE: single-balloon enteroscopy

## Discussion

In our study, SBE was determined to be a useful and safe procedure with a high diagnostic rate, high intervention rate, and low complication rate for the management of small bowel diseases. In addition, SBE is easier to manipulate than DBE, exhibits shorter operative times, and can be performed by a single technician. The SBE procedure is not only convenient for the operator, but also has good safety, as shown in the current study, with experienced operators [8]. In this study, many kinds of small intestinal lesions were diagnosed and treated with SBE.

According to the limited published data regarding SBE, the diagnostic yield ranges from 58% to 70% [5,9–11]. A recent meta-analysis by Lipska et al. comparing SBE and DBE showed no significant differences in diagnostic yield, therapeutic yield, insertion depth, procedure time, failure rate, and complication rate [5]. However, in addition to the experience of the endoscopist, the diagnostic yield of balloon-assisted enteroscopy varies according to restrictions on the indications for SBE. In our study, the diagnostic yield of 61% and the intervention rate of 20.5% (Table 1) were similar to those of previous reports [9]. According to a previous meta-analysis, the diagnostic yield of DBE performed after a previously positive capsule endoscopy is higher than that when DBE is performed in all patients with obscure gastrointestinal bleeding [12]. In addition, capsule endoscopy performed prior to SBE improves both the diagnostic and therapeutic yield of SBE (68% vs. 44%, P = 0.002, and 35% vs. 12%, P = 0.001, respectively) [13]. However, no patients in this study received prior capsule endoscopy, because of economic and facility limitations; therefore, any potential influences on the results of anterograde and retrograde SBE owing to prior use of capsule endoscopy may be excluded in this study.

The complete rate of whole bowel examination was 16.7% in our study, whereas the complete rate of SBE is 11% to 71% in published reports [6]. Although DBE was considered to be superior to SBE with regard to complete small bowel examination [4], this conclusion remains controversial [4,5]. A recent meta-analysis showed no significant differences between DBE and SBE in complete enteroscopy [5]. In addition, published data suggest that although incomplete whole small bowel examination might lead to missed lesions, complete enteroscopy does not appear to affect the diagnostic and therapeutic rate [4,5]. However, further randomized controlled studies are required in order to address this issue.

In our study, the general characteristics did not differ according to whether they were determined using anterograde or retrograde SBE. There were no differences in clinical indications, except for chronic diarrhea, which was more commonly associated with retrograde than with anterograde SBE (Table 1). Anterograde and retrograde SBE also exhibited similar endoscopic findings, with the exceptions of Meckel’s diverticulum, which was limited to observation in retrograde SBE (3.2% [retrograde] vs. 0.0% [anterograde], *P* = 0.081), and a greater detection rate of benign tumors with retrograde SBE (9.5%) than with anterograde SBE (1.1%, *P* = 0.009) (Table 2). Although anterograde SBE allows deeper observation and appears to be more effective than retrograde enteroscopy for evaluation and management of suspected small-bowel disease [7], our findings show no difference between the approaches for diagnostic yield. This similarity might have been caused by the biases of patient selection, indications, and clinical judgments for the route of approach. In addition, the hemostasis rate was higher in anterograde SBE than that in retrograde SBE (22.2% vs. 11.9%, *P* = 0.069, Table 3). Explanations for this phenomenon include a greater detection rate of angiodysplastic lesions with anterograde SBE [7], and the comparative ease of performing endoscopic hemostasis in anterograde SBE when compared to retrograde SBE.

In our study, OGIB was the most common indication for SBE (62.5%). Furthermore, SBE was effective in detecting and confirming the etiology of OGIB, with a diagnosis made in more than half of the patients investigated (65.6%). As demonstrated in past studies and our investigation, OGIB is most commonly associated with angiodysplasia, the majority of which is located at the jejunum. In the present study, the diagnostic yield did not differ between anterograde and retrograde SBE. We postulate that this finding was a consequence of selecting the SBE approach according to the clinical presentation and pre-endoscopy examinations (RBC scan, small intestinal series, and abdominal computerized tomography), as well as the enrolled patients failing to account for the limited number with unidentified suspected lesions.

In our study, we found the most common etiologies among OGIB patients were Meckel’s diverticulum for those under the age of 30 years, non-specific ulcers for those aged 30 to 65 years, and angiodysplasia for those older than 65 years. These results are similar to those reported by previous studies, excluding the observation of non-specific ulcers in those aged 30 to 65 years, whereby previous reports have suggested the most common etiology in this group is tumors. However, tumors were the second most common etiology among this age group in our study.

The major complications of SBE identified in this study included aspiration pneumonia, acute pancreatitis, bleeding, and perforation. The rate of major complication in balloon-assisted enteroscopy is approximately 1–3% [5,14–16]. We determined the overall major complication rate to be 1.5% (1 case of acute pancreatitis after hemostasis and biopsy for duodenal tumor, 1 case of delayed bleeding post-unroofing polypectomy, and 1 case of gut perforation after resection of inverted Meckel’s diverticulum). However, no major complications were identified in patients who underwent diagnostic SBE. In terms of minor complications, there was a significant difference in the rate of post-SBE asymptomatic hyperamylasemia between anterograde and retrograde SBE (13.9% vs. 2.0%). The underlying pathogenesis of hyperamylasemia and acute pancreatitis after SBE remains uncertain. Possible causes of this anterograde SBE-related damage are inflation of the balloon near the ampulla of Vater, the pulling manipulation of anterograde SBE, and the pancreas being mechanically strained while shortening during anterograde enteroscopy [17].

Our study has some limitations. First, clinical data were obtained from a single center and 1 experienced endoscopist. Second, the small number of SBE cases in our study may limit generalizability to the wider population.

Overall, we have demonstrated that SBE is a safe, useful, and easy-to-control tool for the diagnostic treatment of small intestinal disorders. Furthermore, although the outcome and safety of anterograde and retrograde SBE procedures are similar, anterograde SBE has a shorter approach time but a higher rate of asymptomatic hyperamylasemia.
